# Prevalence and associated factors of soil transmitted helminthes among pregnant women attending antenatal care in Maytsebri primary hospital, North Ethiopia

**DOI:** 10.1186/s13104-019-4684-3

**Published:** 2019-10-04

**Authors:** Menasbo Gebru Gebrehiwet, Araya Abrha Medhaniye, Haileselasie Berhane Alema

**Affiliations:** 1 Tigray Health Bureau, Mekelle, Ethiopia; 2 Department of Public Health, College of Health Sciences, Mekelle University, Mekelle, Ethiopia; 3 Department of Public Health, College of Health Sciences, Aksum University, P.O.Box: 298, Aksum, Ethiopia

**Keywords:** Pregnant mother, Primary hospital, Soil transmitted helminthes

## Abstract

**Objectives:**

Soil-transmitted helminthes are among the most common infections worldwide and affect the poorest and most deprived communities. A health facility based cross-sectional study was conducted among pregnant women attending at Maytsebri primary hospital. Data was entered and analysed using SPSS version 20 software. Multivariate analyses were used to identify determinant factors associated with soil transmitted helminthiasis. A total of 448 pregnant women were examined microscopically with a Katokatz technique for parasitological study to each women.

**Results:**

Out of the total 448 pregnant women examined in the primary hospital, 229 (51.5%) women were infected by at least one of the soil transmitted helminthiasis. Hookworm was the most prevalent 179 (78.16%) soil transmitted helminthes infection. Women who had a habit of eating soil were 2.6 times more likely to be infected by soil transmitted helminthes compared to who do not eat soil. Participants who wear shoe always were 95% less likely to be infected by soil transmitted helminthes. Efforts should be done to advance the awareness of women how to prevent soil transmitted helminthes.

## Introduction

Soil-transmitted helminthiasis (STH) is a term referring to a group of parasitic diseases caused by nematode worms that are transmitted to humans by faecally contaminated soil and packages three intestinal parasites hook worm, Ascariasis and trichuriasis [1].

Based on the estimation of WHO more than, 1 billion of the world’s population are chronically infected with soil transmitted helminthes [2]. Beside to this, WHO also reported that, about 40% of disease burden of all tropical infections is due to geohelminths [3]. There are 800 million to one billion people reported to have Ascaris globally and 700–900 million hook worm, 500 million trichuriasis respectively and 300 million suffer associated severe morbidity and even death [3]. In areas of poverty in the developing world Similarly more than 150,000 deaths annually due to helminthes are reported [4]. The global burden of STH infection is estimated at between 5 and 39 million disability-adjusted life years, largely attributable to anemia, stunting, and reduced cognitive development [5].

Globally over 2.5 billion people are still without access to improved sanitation. Over 15% of the population still practice open defecation. In developing regions almost half the population does not have access to sanitary facilities an estimated 1.1 billion people practice open defecation, exposing themselves and their communities to major health risks. In sub-Saharan Africa, only 24% of the rural population was using an improved sanitation facility [6].

In study of Ethiopia, a significant association was found between tuberculosis and intestinal helminthes infection. Some studies show that treatment using soil transmitted helminthes for the adults who have co-infection can increases immunity of individuals [7]. Study from some part of Ethiopia revealed that the prevalence of soil transmitting helminthes was 31.5% [8]. Similar study conducted in Gilgel Gibie, Ethiopia showed that the prevalence of soil transmitted helminthes was found to be (41%). In this study the most common prevalent soil transmitted helminthiasis were hookworm (29.4%), Ascaris lumbricoides (15%) and *Trichuris trichiura* (3.4%) [9].

Reports from the Maytsebri district (study area) showed that there is high maternal morbidity of anemia among pregnant women that consequently resulted with adverse maternal health outcome. This make us to think that this anemia could be due to intestinal parasites. Furthermore, pregnant women do have double burden of health risk that is for themselves and their fetus. Before any interventions made, there should need adequate evidences from the studies. In Ethiopia, particular in Tigray region, there is no evidence showed the prevalence of soil transmitted helminthes among pregnant women. This makes us to focus our study particular in pregnant women that will help to generate evidence to intervene the problem.

Most of the parasitic infection burden is attributable to poor sanitation where, there high prevalence of worm infestations causing contributes to the high maternal complications [10]. Reports from the study area district show that there are poor sanitation and hygienic environment. Moreover, there are only limited studies were conducted on the status of soil transmitted helminthes and no study was conducted in the study area. Therefore, this study was aimed to assess the prevalence of soil transmitted helminthes and associated factors among pregnant women attending antenatal care in Maytsebri primary hospital, Tigray, North Ethiopia.

## Main text

### Methods

#### Study settings

Health facility based cross-sectional study design was employed to assess the prevalence of and associated factors of soil transmitted helminthes among pregnant women in Maytsebri district hospital, north Ethiopia. The primary hospital is located in Tselemti district, North part of Ethiopia. The district was found 386 km from Mekelle capital of the region and 1180 km capital of Ethiopia with total population of 138,858 and comprises of 70,109 males and 68,750 females. Among the reproductive age woman more than 5333 were estimated to be pregnant. The district has one primary hospital, 5 health centers and 21 health posts that render antenatal care services for pregnant women. The primary hospital provides antenatal care service for more than 4500 pregnant women annually. All pregnant women who were attending antenatal clinic at Maytsebri primary Hospital were eligible for the study.

#### Sample size determination and sampling procedure

Sample size was determined using double population proportion with assumptions of 95% confidence interval, percent of exposed outcome (P1) = 50 and percent of unexposed outcome (P2) = 52.2 [8], odds ratio 2.3, and 0.05 level of significance. Through this assumptions, the sample size was calculated using EPI info version 7 software. And total of 448 sampled pregnant women included in the study.

A systematic random sampling method was employed to include 448 study participants. Considering the number of pregnant women who attended ANC in the primary hospitals in 6 months prior to the data collection time, the average number of pregnant women expected to attend ANC per month in the primary hospital was estimated. Finally, every 3rd pregnant mother who visited ANC was recruited until the allocated sample was reached. Data was collected using a semi-structured questionnaire which contained questions related to socio-demographic characteristics, behavioral habits, and environmental conditions. Each participant was provided with labeled screw capped stool container and informed on how to collect stool samples. The collected stool specimens were checked for parasite of helminthes by the Kato-Katz technique within 30 min so as to identify easily the parasites. According to Ethiopian public health institution (EPHI) recommendation, for soil transmitted helminths community diagnosis study the preferred methods when the prevalence considered high is Kato-Katz technique. So, in this study to declare the prevalence of the parasite a single Kato-Katz stool sample was done. For the purpose intervention the prevalence was enough hence what ever the parasitic load the management is similar. That is why the intensity of the parasitic infection was not determined.

#### Data entry and analysis

Information recorded in the questionnaire and results collected from laboratory were checked for completeness and consistency, coded and entered into Epi Info 7 then exported to SPSS version 20.0 software package for further analysis. For the analysis of demographic data descriptive statistics was employed. Prevalence of soil transmitted helminthes calculated based on the stool sample results. After variables with a p-value less than or equal to 0.2 were entered into multivariate logistic regression to identify independent risk factors for the occurrence of STH among pregnant women. p-value < 0.05 was considered significantly associated with soil transmitted helminthes.

#### Data quality assurance

The data collection tool was pre tested to assess its clarity and ambiguity. Some skip pattern was corrected and questions difficult to ask were rephrased. Training was given to data collectors and supervisors for 1 day focusing on understanding the research question, approaching participants, and sampling procedures. The tool was discussed in detail for every question and data collectors demonstrate data collection procedures and techniques. All filled questionnaires were checked daily for completeness, accuracy and consistency by supervisors and the principal investigator and necessary corrections were made. Moreover, stool specimen handling and transportation within specified time to laboratory, orientation and instruction was given to study participants.

### Results

A total of 448 pregnant women were included in the study and 100% response rate was obtained. The mean age of the study participants were 24.6 (± 5.7) year (Table 1).

**Table 1:** Socio demographic characteristics of pregnant women examined for STH in Maytsebri primary hospital, Tigray, Ethiopia, 2018

S. no	Variables	Frequency	Infection of soil transmitted helminthes (n = 229)
1	Age of the respondents		
	18–24 years old	243 (54.2%)	127 (55.5%)
	25–34 years old	172 (38.4%)	85 (37.1%)
	35–44 years old	32 (7.1%)	17 (7.4%)
	> 45	1 (0.2%)	0 (0)
2	Residence		
	Urban	200 (45%)	94 (41.0%)
	Rural	248 (55%)	135 (59.0%)
3	Educational status of the respondents		
	Not attended formal education	247 (55.1%)	128 (55.9%)
	Primary school	119 (26.6%)	67 (29.3%)
	Secondary school	50 (11.2%)	23 (10.0%)
	Diploma and above	32 (6.9%)	11 (4.8%)
4	Occupational status of the respondents		
	Farmer	142 (31.7%)	78 (34.0)
	Daily laborer	19 (4.3%)	8 (4.5%)
	Merchant	49 (10.9%)	24 (10.5%)
	House wife	190 (42.4%)	96 (41.9%)
	Government employee	48 (10.7%)	23 (10.1%)
5	Trimester of pregnancy		
	First trimester	125 (27.9%)	63 (27.5%)
	Second trimester	141 (31.5%)	72 (31.5%)
	Third trimester	182 (40.6%)	94 (41.0%)
6	Gravida		
	Primigravid	145 (32.4%)	76 (33.2%)
	Multigravid	303 (67.6%)	153 (66.8%)

The result of the study indicated that among the study participants, 220 (49.1%) women didn’t have latrines and only 228 (50.9%) women were with latrine. From women with latrine, 207 (90.7%) women were utilized. Among the latrines, 123 (54%) were unclean and 105 (46%) latrines were neat and clean. From the 228 functional latrines 132 (58%) have no hand washing facilities. Among the study subjects, 61 (22.3%) women used water and soap to wash their hands after any procedures. From of these women only 24 (39.4%) women had the habit of washing hands regularly, but 37 (60.6%) others had habit of washing hands sometimes. In this study, the prevalence of soil transmitted helminthes was 51%. The most common prevalent parasites were hookworm 179 (78.2%), *Ascaris* 57 (24.9%), and *Trichuriasis* 5 (2.2%).

#### Risk factors associated with soil transmitted helminthes

Variables including residence, educational status, place of cooking, having latrine, hand washing basin in nearby latrine, disposal of child’s feces, using soap during latrine, wearing shoes, swimming, eating soil, refuse disposal, and health education were significantly associated with soil transmitted helminthes, in bivariate logistic regression analysis with p-value less than 0.2. Then after controlling the confounders in multivariable logistic regression model, variables: wearing shoes and eating soil were factors significantly associated with soil transmitted helminthes. Women who had no habit of hand wash using water and soap after any procedure were 5 times more likely [AOR = 5 (95% CI 1.04–26.4)] to have the soil transmitted helminthes infection compared to those who had practice of washing hands using water and soap. Pregnant women who have habit of wearing shoes were 95% less likely [AOR = 0.05 (95 CI 0.014–0.186)] to be infected by soil transmitted helminthes than who do not wear shoes. With regard to eating soils women who had the habit of eating soil (Geophagia women) during pregnancy were 2.6 times more likely [AOR = 2.6 (95% CI 1.09–6.09)] to have soil transmitted helminthes compared to those do not have habit of soil eating (Table 2).

**Table 2:** Factors associated with soil transmitted helminthes among pregnant women in Maytsebri primary hospital, Tigray, Ethiopia 2018

Variables	Category	STHs		COR (95% CI)	AOR (95% CI)
		Yes	No		
Washing hands after and procedures	Regular	22	31	1	1
	Sometimes	38	57	1.065 (0.54–2.1)	23 (3.7–146.6)
Hand washing using water and soap after any procedure & contact	Always	12	14	1	1
	Sometimes	6	46	6.6 (2.09–20.7)	5 (1.04–26.4)*
Practice of wearing shoes	Yes	129	204	0.095 (0.05–0.2)	0.05 (0.014–0.186)*
	No	100	15	1	1
Frequency habit of wearing shoes	Frequently	88	132	1	1
	Infrequently	41	72	1.17 (0.73–1.87)	0.704 (0.36–1.38)
Habit and practice of swimming	Yes	37	46	0.72 (0.449–1.17)	0.05 (0.015–0.18)
	No	192	173	1	1
Practice and habit of eating soil	Yes	29	17	1.72 (0.918–3.2)	2.57 (1.09–6.0)*
	No	200	202	1	1

* Statistically significant at α < 0.05

### Discussion

The study was focused on prevalence and associated factors of soil transmitted helminthes among pregnant women attending ANC in Maytsebri primary hospital. The prevalence of soil transmitted helminthes in the study was 51%. This finding was higher compared to the study conducted in East Nigeria which was (32.4%) [11], Ibadan (43.4%) [12], Kenya (34%) [13], Ethiopia, Gilgel Gibie (41%) [9], Felege Hiwot (31.5%) [8] and Kitale district hospital, Kenya (13.8%) [3] but almost similar with the finding of Lambarene Gabon (49%) [14]. The possible reasons could be due to low socio-economic status of the community and also there is low latrine construction and utilization which contribute large for soil transmitted helminthes infection burden. And this high prevalence of helminthic parasite in this area could also be due to poor personal hygiene and poor environmental sanitation practices.

The variables associated with soil transmitted helminthes were washing of hands with water and soap, eating of soil and wearing of shoe.

Pregnant women who washed their hands using water and soap sometimes were high risk to be infected by soil transmitted helminthes than those wash their hand regularly using water and soup. This finding was similar with the study conducted in Butajira that seldom washing of hands using soap users were more likely to maternal soil transmitted helminthes infections [15]. The possible justification could be due to the reason of socio-economic differences and lack of awareness on the method of the parasitic transmission. Beside to this, it might be due to inadequacy of water supply and habit of cleaning dirt particles despite trial to use soap frequently is limited.

The study also revealed that women who wearied shoes frequently were less likely to be infected by soil transmitted helminthes as compared to the women who didn’t wear shoes. This result is inline with meta-analysis and systematic review [5], study conducted in Nigeria [12]. This may be reasoned as, those who wear properly and regularly shoes have less chance to be contaminated with infected mud during food eating and water drinking. But those who do not wear shoe regular have a probability of carrying contaminated soil through hands and legs to home and easily contaminate which lead to soil transmitted helminthes infection. This is what happened in most of rural set-up where poor environmental sanitation is observed which attributed to parasitic infection.

This study showed that women who had practice and habit of soil eating during pregnancy were more likely to affected by soil transmitted helminthes when compared to the women who do not have practice and habit of eating soil. This finding was in agreement with studies done in Ethiopia [8, 9]. This may be due to the fact that women during pregnancy have urge and craving soil to eat and this is mostly accepted by the community. Most pregnant women eat soils for varied reasons, some pregnant women preference the soil for its texture of while others for taste. Furthermore, this can be also associated with food taboos and lack of education.

### Conclusion

The prevalence of soil transmitted helminthes in the study area was high. The most common identified parasites were: hookworm, Ascaris lumbricoides and *Trichuris trichiura.* The determinant factors of soil transmitted helminthes were; washing of hands using water and soup, habit of eating soils and wear of shoe. Guidelines should develop for the implementation of routine stool examination screening procedure for all pregnant women attending antenatal care, so that mass management can be offered to all pregnant women where identified high prevalence of soil transmitted helminthes.

## Limitations

The study was facility based that study participants come from different corner of the district which make difficult to generalize the findings of the study. We suggest for future investigations that studies should be community based so that it can intervene easily for specific population.

## Data Availability

The datasets generated and analyzed during this study were included in the main document of this manuscript and available from the corresponding author on reasonable request.

## Abbreviations

AOR: adjusted odds ratio
COR: crude odds ratio
CI: confidence interval
KM: kilo meter
SPSS: statistical package for social sciences
STH: soil transmitted helminthes
WHO: World Health Organization

## References

[1] Montresor A, Crompton DWT. Guidelines for the evaluation of soil-transmitted helminthiasis and schistosomiasis at community level ministry of health and welfare. Government of Japan. 1998.

[2] WHO (2012). Eliminating soil-transmitted helminthiases as a public health problem.

[3] Wekesa AW, Mulambalah CS, Muleke CI, Odhiambo R (2014). Intestinal helminth infections in pregnant women attending antenatal clinic at Kitale District Hospital, Kenya. J Parasitol Res.

[4] Peter JH, Bundy DAP (2006). Disease control priorities in developing countries.

[5] Strunz EC, Addiss DG, Stocks ME, Ogden S, Utzinger J, Freeman MC (2014). Sanitation, hygiene, and soil-transmitted helminth infection: a systematic review and meta-analysis. PLoS Med.

[6] Yimam YT, Gelaye KA, Chercos DH (2014). Latrine utilization and associated factors among people living in rural areas of Denbia district Northwest Ethiopia. Pan Afr Med J.

[7] Walson JL, Otieno PA, Mbuchi M, Richardson BA, Lohman-Payne B, Macharia SW, Overbaugh J, Berkley J, Sanders EJ, Chung M, John-Stewart GC (2008). Albendazole treatment of HIV-1 and helminthes co-infection: a randomized, double blind, placebo-controlled trial. AIDS.

[8] Derso A, Nibret E, Munshea A (2016). Prevalence of intestinal parasitic infections and associated risk factors among pregnant women attending antenatal care center at Felege Hiwot Referral Hospital, northwest Ethiopia. BMC Infect Dis.

[9] Getachew M, Tafess K, Zeynudin A, Yewhalaw D (2013). Prevalence soil transmitted helminthiasis and malaria co-infection among pregnant women and risk factors in Gilgel Gibe dam Area, Southwest Ethiopia. BMC Res Notes.

[10] Fuseinisup G, Edohsup D, Kalifasup BG, Hamidsup AW, Knight D (2010). Parasitic infections and anaemia during pregnancy in the Kassena-Nankana district of Northern Ghana. J Public Health Epidemiol.

[11] Dimejesi IB, Umeora OU, Egwuatu VE (2014). Prevalence and pattern of soil-transmitted helminthiasis among pregnant women in a tertiary health facility, southeast Nigeria. Afr J Med Health Sci.

[12] Alli JA, Okonko IO, Kolade AF, Nwanze JC, Dada VK, Ogundele M (2011). Prevalence of intestinal nematode infection among pregnant women attending antenatal clinic at the University College Hospital, Ibadan, Nigeria Pelagia. Adv Appl Sci Res.

[13] McClure EM, Meshnick SR, Mungai P, Malhotra I, King CL, Goldenberg RL, Hudgens MG, Siega-Riz AM, Dent AE (2014). The association of parasitic infections in pregnancy and maternal and fetal anemia: cohort study in coastal kenya. PLoS Negl Trop Dis..

[14] Adegnika AA, Ramharter M, Agnandji ST, Ateba Ngoa U, Issifou S, Yazdanbahksh M, Kremsner PG (2010). Epidemiology of parasitic co-infections during pregnancy in Lambarene, Gabon. Trop Med Int Health.

[15] Belyhun Y, Medhin G, Amberbir A, Erko B, Hanlon C, Alem A, Venn A, Britton J, Davey G (2010). Prevalence and risk factors for soil-transmitted helminth infection in women and their infants in Butajira, Ethiopia: a population based study. BMC Public Health.

